# A new transport phenomenon in nanostructures: a mesoscopic analog of the Braess paradox encountered in road networks

**DOI:** 10.1186/1556-276X-7-472

**Published:** 2012-08-22

**Authors:** Marco Pala, Hermann Sellier, Benoit Hackens, Frederico Martins, Vincent Bayot, Serge Huant

**Affiliations:** 1 IMEP-LAHC, Grenoble INP, Minatec, BP 257, Grenoble, F-38016, France; 2 Institut Néel, CNRS and Université Joseph Fourier, BP 166, Grenoble, F-38042, France; 3 IMCN/NAPS, UCLouvain, 2 Chemin du Cyclotron, Louvain-la-Neuve, B-1348, Belgium

**Keywords:** Braess paradox, Mesoscopic physics, Congested networks, Scanning gate microscopy

## Abstract

The Braess paradox, known for traffic and other classical networks, lies in the fact that adding a new route to a congested network in an attempt to relieve congestion can degrade counterintuitively the overall network performance. Recently, we have extended the concept of the Braess paradox to semiconductor mesoscopic networks, whose transport properties are governed by quantum physics. In this paper, we demonstrate theoretically that, alike in classical systems, congestion plays a key role in the occurrence of a Braess paradox in mesoscopic networks.

## Background

Adding a new road to a congested road network can paradoxically lead to a deterioration of the overall traffic situation, i.e., longer trip times for individual road users, or, in reverse, blocking certain streets in a complex road network can surprisingly reduce congestion [1]. This counterintuitive behavior has been known as the Braess paradox [2,3]. Later extended to networks in classical physics such as electrical or mechanical networks [4,5], this paradox lies in the fact that adding extra capacity to a congested network can degrade counterintuitively its overall performance.

**Figure 1:**
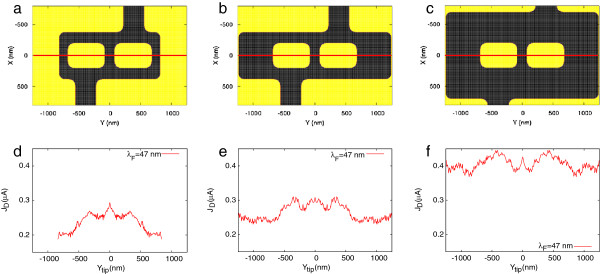
**Evidence for the key role of network congestion in occurrence of mesoscopic Braess paradox.** (**a**, **b**, **c**) Network geometries: all networks have a central (additional) branch of 160-nm width and 320-nm wide openings. In (a), *W* = 140 nm, *L* = 180 nm. In (b), *W* = 560 nm, *L* = 180 nm. In (c), *W* = 560 nm, *L* = 500 nm. (**d**, **e**, **f**) The current transmitted through the networks as a function of the tip position scanned along the median lines of the networks (red lines in (a, b, c)). The source-drain voltage applied to the networks is *V*_ds_ = 1 mV, and the potential applied to the tip is −1 V (see text). Fermi wavelength (*λ*_F_) = 47 nm, *T* = 4.2 K.

Known so far in classical networks only, we have recently extended the concept of the Braess paradox to the mesoscopic world [6]. By combining quantum simulations of a model system and scanning gate microscopy [7-11], we have discovered that an analog of the Braess paradox can occur in mesoscopic electron networks, where transport is governed by quantum mechanics. To explore the possibility of a mesoscopic Braess paradox, we had set up a simple two-path network in the form of a hollow rectangular corral connected to a source and a drain via two openings, with dimensions such that the embedded two-dimensional electron gas (2DEG) is in the ballistic and coherent regimes of electron transport at 4.2 K. The short wires in the initial corral, Figure 1a, were narrower than the long wires in order to behave as congested constrictions for propagating electrons (see below). Branching out this basic network by adding a central wire as shown in Figure 1a opens an additional path to the electrons. Also, we have used scanning gate microscopy [7-11] to partially block by local gate effects the electron transmission through this additional path. Doing so should intuitively result in a decreased total current transmitted through the device since one electron path partly loses efficiency, but we counterintuitively found, both numerically and experimentally, that it is exactly the opposite behavior that can actually take place [6]. 

A key ingredient in the occurrence of classical Braess paradoxes is network congestion. Our previous work was made on a congested mesoscopic network, and it indeed exhibited a marked paradoxical behavior. In this letter, we study numerically in more detail the effect of congestion by simulating three rectangular corrals of different dimensions, i.e., different degrees of congestion. We show that releasing congestion considerably relaxes the paradoxical behavior. Simulations of the spatial distribution of the current density inside the networks for different positions of the local gate help to interpret our predictions in terms of current redistribution inside the network.

## Methods

### Theoretical details

The three simulated networks are shown in Figure 1a,b,c. The narrowest network in Figure 1a is nearly identical to the network simulated in our previous work [6], apart from slightly larger openings (320 nm instead of 300 nm). Its dimensions are chosen such that the electron flow is congested. Indeed, in a system where electrons can be backscattered solely by the walls defining the structure geometry, a sufficient condition to reach congestion is obtained when the number of conducting modes allowed by internal constrictions is smaller than the number of conducting modes in the external openings, which implies 2 *W* < *W*_0_ , where *W* and *W*_0_ denote the widths of the lateral arms (both of the same width) and of the external openings (of equal widths too), respectively. In turn, increasing *W* such that 2 *W* > *W*_0_ , as shown in Figure 1b, progressively relaxes congestion since all conducting modes injected by the openings can be admitted in the lateral arms. Starting from the network of Figure 1b, we will further relax the congestion by increasing the widths *L* of the horizontal long arms, as shown in Figure 1c.

The transport properties of these structures are simulated within an exact numerical approach based on the Keldysh Green’s function formalism. A thermal average is performed around the Fermi energy *E*_F_ at the temperature *T* = 4.2 K. We adopt a mesh size of Δ*x* = Δ*y* = 2.5 nm. The Green’s function of the system is computed in the real space representation that allows us to take into account all possible conducting and evanescent modes. Moreover, in order to reduce the computational time and memory requirements, we exploit a recursive algorithm, which is based on the Dyson equation [6,9].

In this framework, the current densities along the *x*-axis (transport direction) and the *y*-axis (transverse direction) between two adjacent nodes read as follows:

(1) Ji,i+1;k,k=−4eh∫dωReHi,i+1;k,kGi+1,i;k,k<ω,

(2) Ji,i;k,k+1=−4eh∫dωReHi,i;k+1,kGi,i;k+1,k<ω,

where *H*_*i,i';k,k'*_ represents the Hamiltonian discretized on the local basis, and *G*^<^_*i,i';k,k'*_(*ω*) is the ‘lesser-than Green’s function’ [9] in the real space representation and energy domain.

The tip-induced potential is simulated by considering a point-like gate voltage of −1 V placed at 100 nm above the 2DEG, which corresponds to a lateral extension of ≈ 400 nm for the tip-induced potential perturbation at the 2DEG level.

## Results and discussion

### The key role of congestion in the network

Figure 1d,e,f shows the current flowing through the structures depicted in Figure 1a,b,c, respectively, as a function of the tip position scanned along the median lines (red lines). Figure 1d shows the occurrence of an analog of the classical Braess paradox in a congested mesoscopic network as a distinctive current peak centered at *Y*_tip_ = 0 nm. When the tip-induced potential closes the central wire connecting the two openings in Figure 1a, the current is counterintuitively increased. However, Figure 1e,f shows that as soon as the condition for congestion is relaxed, allowing a larger number of conducting channels to propagate in the region inside the structure, the paradox disappears, and the total current exhibits a maximum when the tip is placed over the two antidots.

**Figure 2:**
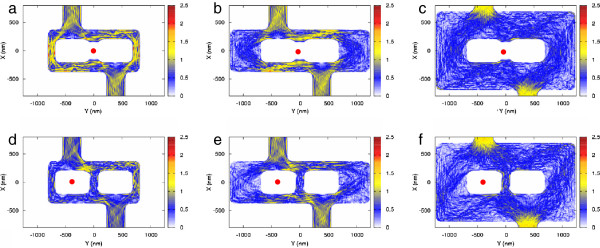
**Current redistribution in the mesoscopic networks.** All figures depict contour plots of the spatial distribution of the current density. (**a**, **b**, **c**) The tip, marked by a red dot, is positioned above the middle of the networks, i.e., above the center of the additional arm. (**d**, **e**, **f**) The depleting tip is positioned above the center of the left-hand side antidot. Fermi wavelength (*λ*_F_) = 47 nm, *V*_ds_ = 1 mV, and *T* = 4.2 K.

In order to microscopically study this behavior, we have simulated in Figure 2 the spatial distribution of the absolute value of the current |*J*| inside the three structures for *Y*_tip_ = 0 nm and *Y*_tip_ = −400 nm. When comparing Figure 2a and Figure 2d for the congested structure, we can notice that the opening of a third central wire connecting the contacts has a twofold effect. The first consequence is to create a direct connection between the source and the drain, which should positively contribute to the total current flowing through the system. The second one is to generate alternative paths that trap electrons in the central region and should promote a longer stay inside the network. We believe that this second effect is the one responsible for the decrease of the total current as long as the third wire is opened. The comparison of Figure 2a and Figure 2d is indeed very instructive, and in particular, the behavior of the current through the right path paradoxically decreases while the depleting tip moves away. This behavior clearly indicates that the current contribution of trapped electrons around the right antidot compensates partially the initial current. This effect is only partly replicated in the networks of Figure 1b,c, whose current redistributions are shown in Figure 2b,e and Figure 2c,f, respectively. In these cases, the reopening of the third wire, obtained by placing the tip over the antidot, induces a number of new internal paths, which are small compared to the large number of semiclassical trajectories already present in the lateral arms. Therefore, the closing of the central path implies only a small current increase in Figure 1e,f around the position *Y*_tip_ = 0 nm, which is not sufficient to overcome the current at *Y*_tip_ = −400 nm.

### The robustness of the paradox

**Figure 3:**
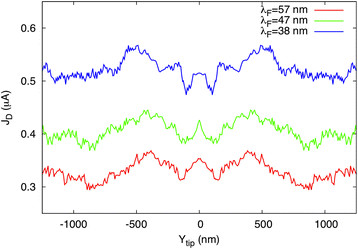
**Robustness of the results.** The current transmitted through the network of Figure 1c as a function of the tip position scanned along the median line (red lines in Figure 1c) for three different Fermi wavelengths (*λ*_F_ = 57, 47, and 38 nm). The potential applied to the tip is −1 V, *V*_ds_ = 1 mV, and *T* = 4.2 K.

Finally, in order to test the robustness of our results, we simulated the non-congested structure of Figure 1c at different Fermi wavelengths (*λ*_F_ = 57, 47, and 38 nm). This is shown in Figure 3. The behavior of the three curves is qualitatively very similar: they present two regions of maximum current when the gated tip is placed over the two antidots, allowing the passage of electrons through the central path, but they also show a local increase in current around *Y*_tip_ = 0, when the tip closes the central path. This is a signature that the mechanism responsible for the occurrence of the paradox in the congested structure of Figure 1a, even if still present, is not predominant with respect to the direct coupling between the two contacts provided by the third wire.

## Conclusions

In this letter, we have studied the geometric conditions of mesoscopic networks for the occurrence of a quantum analog of the Braess paradox, known previously for classical systems only. By analyzing the spatial distribution of current density in different structures, we have shown that congested structures are the most suitable geometries to the occurrence of such a counterintuitive phenomenon. This is reminiscent to what is known for the classical paradoxes, in particular, for the historic road-network Braess paradox.

## Competing interests

The authors declare that they have no competing interests.

## Authors’ contributions

MP performed all of the simulations. SH initiated the work and presented the talk at the ICSNN 2012. MP and SH wrote the paper. MP, HS, BH, FM, VB, and SH all animated the discussions on the Braess paradox, extensively discussed the results, and proofread the article. All authors read and approved the final manuscript.

## References

[B1] Youn H Gastner MT Jeong H Price of anarchy in transportation networks: efficiency and optimality transport Phys Rev Lett 2008 101 128701 18851419 10.1103/PhysRevLett.101.128701

[B2] Braess D Uber ein paradoxon der verkehrsplanung Unternehmensforschung 1968 12 258 268

[B3] Braess D Nagurney A Wakolbinger T On a paradox of traffic planning Transp Sci 2005 39 446 450 10.1287/trsc.1050.0127

[B4] Cohen JE Horowitz P Paradoxical behavior of mechanical and electrical networks Nature 1991 352 699 701 10.1038/352699a0

[B5] Penchina CM Penchina LJ The Braess paradox in mechanical, traffic, and other networks Am J Phys 2003 71 479 482 10.1119/1.1538553

[B6] Pala MG Baltazar S Liu P Sellier H Hackens B Martins F Bayot V Wallart X Desplanque L Huant S Transport inefficiency in branched-out mesoscopic networks: An analog of the Braess paradox Phys Rev Lett 2012 108 076802 22401236 10.1103/PhysRevLett.108.076802

[B7] Hackens B Martins F Ouisse T Sellier H Bollaert S Wallart X Cappy A Chevrier J Bayot V Huant S Imaging and controlling electron transport inside a quantum ring Nature Phys 2006 2 826 830 10.1038/nphys459

[B8] Martins F Hackens B Pala MG Ouisse T Sellier H Wallart X Bollaert S Cappy A Chevrier J Bayot V Huant S Imaging electron wave functions inside open quantum rings Phys Rev Lett 2007 99 136807 17930624 10.1103/PhysRevLett.99.136807

[B9] Pala MG Hackens B Martins F Sellier H Bayot V Huant S Ouisse T Local density of states in mesoscopic samples from scanning gate microscopy Phys Rev B 2008 77 125310

[B10] Hackens B Martins F Faniel S Dutu CA Sellier H Huant S Pala MG Desplanque L Wallart X Bayot V Imaging Coulomb islands in a quantum Hall interferometer Nat Commun 2010 1 39 20975700 10.1038/ncomms1038

[B11] Sellier H Hackens B Pala MG Martins F Baltazar S Wallart X Desplanque L Bayot V Huant S On the imaging of electron transport in semiconductor quantum structures by scanning-gate microscopy: successes and limitations Semicond Sci Technol 2011 26 064008 10.1088/0268-1242/26/6/064008

